# Effect of a *Bacillus velezensis* and *Lysinibacillus fusiformis*-based biofertilizer on phosphorus acquisition and grain yield of soybean

**DOI:** 10.3389/fpls.2024.1433828

**Published:** 2024-08-23

**Authors:** Luciana Cristina Vitorino, Elias José da Silva, Marilene Silva Oliveira, Isabella de Oliveira Silva, Lorraine da Silva Santos, Maria Andréia Corrêa Mendonça, Thais Cristina Sousa Oliveira, Layara Alexandre Bessa

**Affiliations:** ^1^ Laboratory of Agricultural Microbiology, Federal Institute Goiano, Rio Verde, GO, Brazil; ^2^ Simple Verde Bio-Industry, Simple Agro Corporation, Rio Verde, GO, Brazil; ^3^ Laboratory of Biochemistry and Genetics, Federal Institute Goiano, Rio Verde, GO, Brazil; ^4^ Laboratory of Metabolism and Genetics of Biodiversity, Federal Institute Goiano, Rio Verde, GO, Brazil

**Keywords:** endophytic bacteria, insoluble phosphorus, phosphate mineralization, rhizobacteria, phosphate solubilization

## Abstract

**Introduction:**

Phosphate-solubilizing bacteria that function through acidification (organic acid synthesis) or mineralization (production of enzymes such as phytase and phosphatases) have been explored as a biotechnological alternative to enhance plant access to phosphorus (P) retained in organic and inorganic forms in agricultural soils. This study tested the hypothesis that applying a biofertilizer composed of a recognized phosphate-solubilizing bacterium (*Bacillus velezensis* – endophytic strain BVPS01) and an underexplored plant growth-promoting bacterium (*Lysinibacillus fusiformis* – endophytic strain BVPS02) would improve the growth and grain yield of *Glycine max* L. plants.

**Methods:**

Initial in vitro tests assessed the functional traits of these bacteria, and a mix of strains BVPS01 and BVPS02 was produced and tested under field conditions to evaluate its agronomic efficiency.

**Results:**

The results confirmed the hypothesis that the tested biofertilizer enhances the agronomic performance of *G. max* plants in the field. The *B. velezensis* strain (BVPS01) was found to be more effective than the *L. fusiformis* strain (BVPS02) in solubilizing phosphates via the phosphatase enzyme production pathway, indicated by the expression of the *phoC* and *phoD* genes. In contrast, *L. fusiformis* was more effective in solubilizing phosphates through organic acid and phytase-related pathways, in addition to synthesizing indole-3-acetic acid and increasing the mitotic index in the root meristem of *G. max* plants. These strains exhibited biological compatibility, and the formulated product based on these rhizobacteria enhanced root development and increased the number of nodules and flowers, positively affecting 1000-grain weight, grain yield, and grain P content.

**Discussion:**

Thus, the tested biofertilizer demonstrated potential to improve root growth and increase both the yield and quality of soybean crops, making it a sustainable and low-cost strategy.

## Introduction

Over the past few decades, an agriculture highly dependent on the use of phosphate fertilizers has resulted in accumulation of large amounts of phosphorus (P) in agricultural soils, with surface soil P contents ranging from 50 to 3,000 mg kg^-1^ (Zhu et al., 2018). Nevertheless, the demand and use of phosphate fertilizers to maintain a continuous supply of P to plants has been increasing over time (Rawat et al., 2021). This is because only 0.1% of total P is available for plant uptake due to precipitation with cations in the soil, immobilization, adsorption, and interconversion to organic form (Kishore et al., 2015). Thus, phosphate-solubilizing and -mineralizing bacteria have been used as a biotechnological alternative for improving plant access to the P retained in agricultural soils.

Concerns about P availability for agricultural crops are justified because P is the second most demanded macronutrient by plants after nitrogen. Studies have shown that P is involved with the main metabolic processes in plants, including cell division, energy production, macromolecule biosynthesis, cell membrane integrity, signal transduction, and photosynthesis (Hawkesford et al., 2023). Additionally, P participates in plant respiration processes (Khan et al., 2010) and nitrogen fixation in legume species (Mitran et al., 2018). Concerns about P availability are even more significant when considering subtropical and tropical soils, which are highly weathered and consequently acidic and rich in free Fe and Al, such as Ferralsols (Ultisols) (Juo and Franzluebbers, 2003). These soils typically have low P bioavailability due to P fixation to free cations.

Phosphate-solubilizing rhizobacteria (PSR) can make insoluble phosphates available in the soil by several mechanisms, including secretion of organic acids, production of enzymes such as phosphatases and phytases, and excretion of siderophores that can chelate metal ions and form complexes, making phosphates available for plant uptake. Organic acid production, mainly citric, oxalic, and succinic acids, is probably the primary solubilization pathway for the inorganic P fraction in the soil (Adeleke et al., 2017). These acids utilize their hydroxyl and carboxyl groups to chelate cations bound to phosphate, converting it to a soluble form (Lee et al., 2012).

However, the organic P fraction accounts for 20% to 30% of total soil P, and the dissolution of organic phosphates occurs through mineralization processes mediated by enzymes (Kumar and Shastri, 2017). Phytases catalyze the removal of P from phytate (an abundant form of soil organic P), whereas phosphatases promote the dephosphorylation of phosphoesters or phosphoanhydride bonds of organic compounds (Sharma et al., 2013). The phoC and phoD genes encode acidic and alkaline phosphatases secreted by PSR, respectively (Luo et al., 2019; Li et al., 2021).

PSR not only solubilize phosphates, but also promote plant growth and affect crop yield, as they can synthesize phytohormones such as auxins and cytokinins, which affect cell elongation and cytokinesis processes (Sosnowski et al., 2023). Faster mitotic processes lead to plant growth (Perrot-Rechenmann, 2010).

Thus, considering strategies to improve the performance of *Glycine max* plants in the field, the hypothesis raised in this study was that the application of a biofertilizer composed of a phosphate-solubilizing bacterium (*Bacillus velezensis*) and a bacterium less evaluated as a plant growth promoter (*Lysinibacillus fusiformis*) can improve grain yield of *G. max* crops. This hypothesis was initially tested by conducting *in vitro* tests to assess the ability of the strains of *B. velezensis* (BVPS01) and *L. fusiformis* (BVPS02) to solubilize phosphates through pathways associated with organic acid and phytase production and through mineralization processes by expressing the phoC and phoD genes. Indole-3-acetic acid (IAA) production and the effect of bacterial inoculation on the mitotic index in the root meristem of soybean plants were also evaluated. Subsequently, the preliminary data resulting from *in vitro* assays guided the formulation of a mixture of the tested bacterial strains (BVPS01 and BVPS02) for conducting field experiments to evaluate the agronomic efficiency of the biofertilizer.

*B. velezensis* is an aerobic, Gram-positive, endospore-forming bacterium that promotes plant growth. Various strains of this species suppress the growth of microbial pathogens, including bacteria, fungi, and nematodes (Rabbee et al., 2019). Studies have shown that these bacteria can solubilize several phosphate sources, promoting the growth of plants such as wheat, maize, and soybean (Mosela et al., 2022; Afzal et al., 2023). However, *L. fusiformis*, is a bacterium that has been underexplored biotechnologically. Silva et al. (2015) reported that a strain of *L. fusiformis* isolated from *Psychotria hoffmannseggiana* produced high concentrations of cellulase and pectinase enzymes and exhibited antagonistic activity against *Aspergillus carbonarius*. Jamal and Ahmad (2022) highlighted the large quantity of valuable metabolites produced by bacteria of the genus *Lysinibacillus*, with potential applications in agriculture.

Considering the biological compatibility between the *B. velezensis* and *L. fusiformis*, the objective of this study was to formulate a biofertilizer based on strains of these two bacteria for use in soybean crops, based on analysis of the interaction between them on the growth and grain yield of *G. max* plants, and test the performance of this biofertilizer in the field. Therefore, this study contributes with information for a better understanding of the plant-microorganism interaction and the dynamics of P in soils under application PSR. This information can contribute to the development of better strategies for managing agricultural crops and reversing soil decline.

## Materials and methods

### *In vitro* assays

#### Assessment of phosphate solubilization

The phosphate solubilization potential of two isolated bacterial strains (BVPS01 and BVPS02) was evaluated. These strains were obtained from the culture collection of the Simple Verde^®^ (Simple Agro Corporation) and molecularly identified as *Bacillus velezensis* and *Lysinibacillus fusiformis*, respectively.

The phosphate solubilization potential of each bacterial strain was assessed by growing bacterial samples under constant agitation using an orbital shaker (New Nova Técnica NT 712) at 90 rpm for 24 hours at 30°C in 7 mL of liquid GL culture medium (containing 10 g of glucose and 2 g of yeast extract). Subsequently, 3 mL of each culture was aseptically removed to determine the optical density at 600 nm. The optical density of all bacterial samples was adjusted to 0.1 by dilution with sterile saline solution (0.85%). The tests were performed in triplicate; a culture medium absent from inoculum was used as negative control.

The solubilization of CaHPO_4_, AlPO_4_, and FePO_4_ was quantified in liquid medium by inoculating 1 mL of a standardized bacterial culture into 10 mL of liquid GL medium supplemented with 1.26 g L^-1^ of each phosphate source (CaHPO_4_, AlPO_4_, and FePO_4_). The cultures were shaken at 90 rpm at 30°C for 72 hours. The pH was measured after growth and inorganic P content was determined using ascorbic acid colorimetry at 725 nm, as described by Gadagi and Sa (2002). Inorganic P contents found in control samples were subtracted from those found in bacterial samples to determine the solubilization attributed only to the action of bacteria. The pH levels of bacterial samples were subtracted from those of control samples to detect potential acidification.

#### Assessment of phytase activity

The phytase enzyme activity was assessed in both cultures and cell lysates of *B. velezensis* and *L. fusiformis*. The bacteria were grown in a nutrient broth containing 5 g of peptone and 3 g of beef extract adjusted to pH of 6.8 ± 2 for 72 hours at 30°C under agitation at 200 rpm. The cultures were adjusted to an optical density of 0.8 and then centrifuged at 10,000 g for 5 minutes to obtain the bacterial supernatant and pellet. The pellet was resuspended in SDS solution (20%) and centrifuged again to obtain the cell lysate. Subsequently, 150µL of the culture supernatant or cell lysate from each bacterium was incubated with 600µL of 0.2% sodium phytate substrate (w v^-1^) in 0.1 M sodium acetate buffer (pH of 4.8) for 30 minutes at 39°C. The reaction was stopped by adding 750µL of 5% trichloroacetic acid solution (w v^-1^), and free phosphate was determined as described above. The results were compared to a standard curve prepared with inorganic phosphate (K_2_HPO_4_). The data were obtained from quintuplicates, using the nutrient broth as a control.

#### Assessment of relative expression of phoC and phoD genes

The relative expression of the phoC and phoD genes was evaluated in cultures of *B. velezensis* and *L. fusiformis* g grown in GL medium containing the three tested phosphate sources (CaHPO_4_, AlPO_4_, and FePO_4_). The growth medium was prepared as mentioned above; the cultures were maintained at 28°C under agitation at 200 rpm for 48 hours. Subsequently, the samples were lyophilized for 72 hours and used for gene expression assessments. The phosphate sources were evaluated in triplicate. Total RNA was then extracted by freezing the samples in liquid nitrogen and macerating them with pestles; 1 mL of TRIzol reagent (Thermo Fisher Scientic) was added to the material, and extraction was performed according to the manufacturer’s recommendations. The quantity and quality of the extracted RNAs were determined by spectrophotometry using a Nanodrop 2000c (Thermo Fisher Scientific).

Subsequently, 1µg of total RNA from each sample was treated for 30 minutes with DNase (Thermo Fisher Scientific). A 5µL aliquot of each sample was used in reverse transcriptase reactions using the GoScript Reverse Transcription System kit (Promega) in a 10µL reaction, according to the following protocol: 25°C for 5 minutes, 42°C for 60 minutes, and 70°C for 15 minutes. All samples were then amplified in qPCR reactions in triplicate, as follows: 2µL of sample (1:10 diluted cDNA), 5µL of GoTaq qPCR Master mix (Promega), 250 nM of each primer, 0.1µL of CXR (Promega), and 0.9µL of nuclease-free water. The reactions were conducted on a StepOnePlus device (Applied Biosystems). The primers proposed by Fraser et al. (2017) and Sakurai et al. (2008) were used for the phoC and phoD genes, respectively. The Eub gene was used for parameter normalization (Muyzer et al., 1993). The F and R sequences of these primers, as well as the cycling conditions used for amplification, are shown in **Supplementary Table 1**. The amplification data were analyzed using the LinReg PCR software (Ramakers et al., 2003), and N0 data were used for calculating the relative expression of the phoC and phoD genes, using the Eub gene as the normalizer:


$$Expression\;value=\frac{mean\;N0\;(target\;gene)}{mean\;N0\;(reference\;gene)}$$

#### Assessment of indole-3-acetic acid (IAA) synthesis

IAA production was determined using the colorimetric method described by Gordon and Weber (1951). An aliquot of 1 mL of each standardized bacterial culture was inoculated into 9 mL of nutrient broth supplemented with 100μL of tryptophan. The cultures were incubated at 30°C in the dark for 72 hours and then centrifuged at 16,000 × g for 5 minutes at 4°C to separate the supernatant from the bacterial cells; then 1 mL of the supernatant from each isolate was transferred to a test tube, and 1 mL of Salkowski’s reagent (1.875 g of FeCl_3_.6H_2_O, 100 mL of H_2_O, and 150 mL of H_2_SO_4_) was added to each test tube. The tubes were kept in the dark for 20 minutes and then read in a spectrophotometer at 530 nm. The microorganism’s ability to synthesize IAA was demonstrated by the reddish-pink color of the mixture in the test tubes. Nutrient broth without inoculum was used as a negative control. IAA concentrations were obtained using the equation of the standard calibration curve (Pereira et al., 2012).

#### Assessment of root cell division induction

The effect of the bacterial isolates (BVPS01 and BVPS02) on the mitotic index of soybean root cells was evaluated. The optical density of the bacterial cultures was adjusted to 0.8 after growth in nutrient broth. The strains were inoculated onto soybean seeds using nutrient medium as control. The treatments (seeds treated with BVPS01 or BVPS02 and control seeds) were evaluated at two root development stages (3 and 6 days after inoculation). Each treatment was replicated three times with 25 seeds per replication. The tests were conducted on two sheets of Germitest paper pre-moistened with distilled water in an amount corresponding to 2.5 times the weight of the dry substrate (Brasil, 2009). The resulting rolls were placed in plastic bags and transferred to a BOD incubator (Tecnal, TE-402) at 25°C.

Root apices were collected from the seedlings at 3 and 6 days after inoculation. The mitotic index was determined using the squash technique (Guerra and Souza, 2002). The roots were immersed directly in Carnoy’s fixative (ethyl alcohol and glacial acetic acid at a 3:1 ratio) and stored in a freezer until slide preparation (Chieco and Derenzini, 1999). The root meristematic region was used for the preparation of slides, which were subjected to three 5-minute washes in distilled water, followed by hydrolysis in HCl 5M for 15 minutes at room temperature. Subsequenlty, the slides were washed again with distilled water three times (five minutes each) and stained with Shiff’s reagent (1 g of basic fuchsin, 3 g of potassium metabisulfite, 30 mL of 1M HCL, 200 mL of distilled water, and 0.5 of activated charcoal) for 1 hour at room temperature (Chieco and Derenzini, 1999). Finally, the roots were washed three times again with distilled water.

The meristems were collected and transferred to slides. A 2% orcein solution was applied for squashing the material, followed by the addition of a coverslip to press the material. Five slides were prepared per treatment, and 200 cells per slide were observed. The slides were examined under a light microscope (Olympus, BX61), and the number of cells in different mitotic phases (prophase, metaphase, anaphase, and telophase) was counted. The mitotic index was estimated for the different phases of cell division using the following equation:


$$Mitotic\;index=\frac{Number\;of\;cell\;in\;mitotic\;phase}{Total\;number\;of\;cells}\times 100$$

#### Assessment of compatibility between the isolates

The bacterial strains were grown separately and together in nutrient broth at 28°C under agitation at 200 rpm for 48 hours. Subsequently, the serial dilution technique was used to obtain colonies of the bacteria maintained separately and together, followed by counting of colony-forming units (CFU).

### Statistical analysis of *in vitro* assay data

The data obtained for phosphate solubilization, relative expression of phoC and phoD genes, and IAA synthesis were subjected to normality tests and ANOVA; when the effect of rhizobacteria was significant, the means were compared using Student’s t-test at a 5% significance level to test any contrast between the strains. The data obtained for the mitotic index were compared using Tukey’s test at 5% significance level within each mitotic phase evaluated (prophase, metaphase, anaphase, and telophase).

### Field tests

### Experimental area and implementation

A biofertilizer consisting of a mix of the two phosphate-solubilizing rhizobacteria tested *in vitro* (*B. velezensis* and *L. fusiformis*) was evaluated in field experiments. This biofertilizer was registered as Titânico^®^.

**Figure 1:**
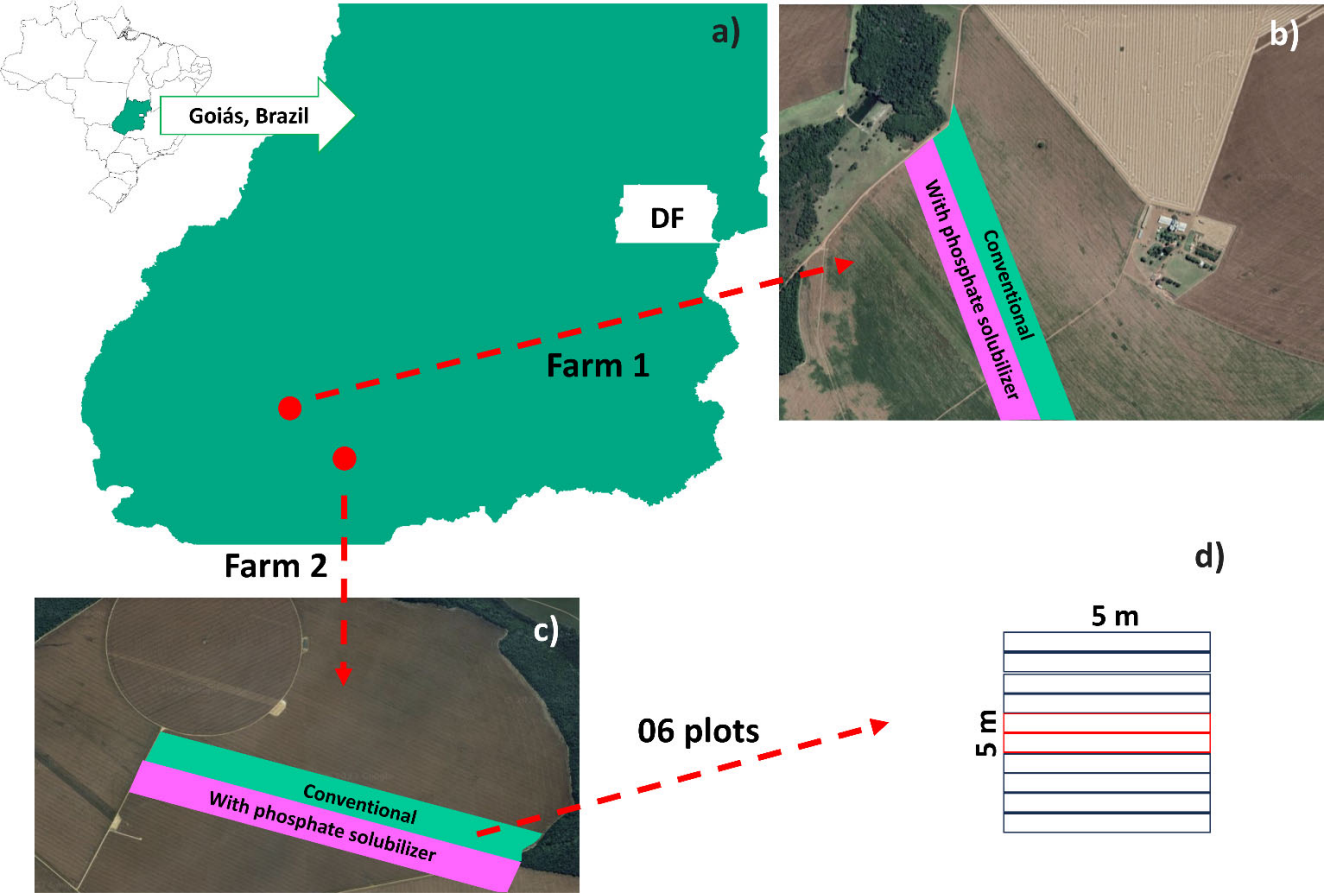
Experimental areas in Goiás, Brazil **(A)** used for growing *Glycine max* crops to evaluate the effect of inoculation with a biofertilizer based on phosphate-solubilizing rhizobacteria (*Bacillus velezensis* and *Lysinibacillus fusiformis*). Experimental area on Farms 1 **(B)** and 2 **(C)** and plants evaluated in six plots of 5 × 5 m randomly arranged in the planting strips; the central red lines in each plot **(D)** were used to evaluate grain yields. Conventional treatment: areas without treatment with the biofertilizer.

The field experiment was established and conducted in two independent experimental areas on farms located in the southwest region of Goiás, Brazil: Fazenda Segredo (Farm 1) at 17°29’11.724”S and 51°27’54.659”W, and altitude of 925 meters; and Fazenda São Francisco (Farm 2) at 18°5’47.339”S and 50°45’38.628”W, and altitude of 767.6 meters (**Figure 1A**). Both experimental areas had the same cropping history, with soybean crops in the summer (October to February) followed by maize crops (March to September). The experiment was conducted during the 2022/2023 crop season, from October 8, 2022 to February 4, 2023. The climate conditions in the region during the experimental period presented mean temperature of 23.09°C, mean relative air humidity of 72.48%, and daily mean rainfall depth of 4.11 mm (**Supplementary Figure 1**).

Three soil samples were randomly collected within and between rows of the plots in each experimental area before implementing the experiments and then sent for chemical and granulometric analysis (**Supplementary Table 2**).

The experimental design consisted of strips with two treatments, one with and one without inoculation with phosphate-solubilizing rhizobacteria (PSR). The treatments were tested side-by-side in six plots randomly distributed within each strip (**Figures 1B, C**). The plots within the strips consisted of ten 5-meter rows spaced 50 cm apart; the eight central rows were considered the evaluation area (**Figure 1D**). The two central rows were maintained until the end of the experiment for grain yield evaluation; 50 cm of each end of the plot were disregarded, resulting in an evaluation area of 20 m^2^.

The experimental areas were maintained under a no-tillage system, using soybean cultivars recommended for the region: 73I75 IPRO (Farm 1) and Voraz IPRO (Farm 2). Soybean seeds were pre-treated with 100 mL of fungicide Maxim Advanced, 200 mL of insecticide Cruizer 350 FS, and 500 mL of insecticide Fipronil per 100 kg of seeds. Soil fertilizers were applied using 165 kg ha^-1^ of the 10–46-00 NPK formulation as basal dressing and 160 kg ha^-1^ of KCl as topdressing

The treatment with the PSR-based biofertilizer was compared to a treatment without using the biofertilizer, representing the conventional management used in the evaluated farms, consisted of applying other microorganisms (commercial products) to the planting furrows; the treatment with PSR-based biofertilizer consisted of combining it to the conventional treatment (**Table 1**). Commercial biological products of the same company (Simple Verde^®^; Simple Agro Corporation) were used in all evaluated areas.

**Table 1:** Treatment and rates used to evaluate the agronomic efficiency of using a biofertilizer based on phosphate-solubilizing rhizobacteria (*Bacillus velezensis* and *Lysinibacillus fusiformis*) in *Glycine max* crops.

Treatment	Microorganisms	Rate (mL ha^-1^)
**Conventional**	*Trichoderma harzianum* *Bradyrhizobium diazoefficiens* *Azospirillum brasilense*	100 175 200
**With phosphate-solubilizing rhizobacteria**	*Trichoderma harzianum* *Bradyrhizobium diazoefficiens* *Azospirillum brasilense* *Bacillus velezensis and Lysinibacillus fusiformis*	100 175 200 100

The treatments were applied to the planting furrows using the Orion agricultural implement system (Orion 3437 Kit) coupled to a tractor that uses a hydraulic system to generate kinetic energy for its operation. The tractor’s rotation was maintained at 1500 rpm and the system was calibrated to apply 40 L ha^-1^ of the solutions. The solutions corresponding to the treatments were prepared for application in a 15-hectare area, consisted of the strip of each treatment.

### Assessment of plant growth, grain yield, and P content in soybean grains

Soybean plants were evaluated at 7, 14, 21, 35, and 119 days after emergence (DAE). Number of nodules, taproot length (cm), shoot length (cm), and root-to-shoot ratio were assessed at 7 DAE. Sampling was carried out with the aid of a garden pickaxe, evaluating 05 plants randomly collected in each plot.

The number of emerged seedlings or plants per linear meter was counted at 14, 21, and 35 DAE in the four meters of the two central rows. Plant vigor was evaluated using a portable canopy vigor-integrity meter (Trimble NDVI Analyzer; GreenSeeker Handheld). The plants reached the Vn/R phenological stage at 135 DAE, when five plants per plot were sampled to evaluate the number of flower buds, number of nodules of 5 to 8 mm diameter, and total number of nodules per plant. The nodule diameter was measured with the aid of a digital caliper.

The two central rows of each plot were harvested manually (119 DAE) using a mechanical thresher to determine grain yield (60-kg bags ha^-1^) and 1000-grain weight (g). Subsequently, grains were sampled to determine P contents (g kg^-1^) using molybdenum blue spectrometry, as described by Da Silva (2009).

### Statistical analyses of field test data

The data obtained for the different biometric parameters and grain yield of the soybean crops were analyzed separately for each farm and subjected to normality tests and ANOVA. When the effect of using the biofertilizer was significant, the means were compared by the Student’s t-test at 5% significance level to test contrasts between the treatments. The exploratory variable and the biometric and grain yield variables were analyzed together by correlation matrix and combined in a principal component analysis (PCA) (one for each farm). Considering that these variables had different units of measurement, the PCA correlations were analyzed using standardized data with a mean of 0 and a standard deviation of 1. The number of components was chosen based on the eigenvalues (>1.0) and explanation of data variance (above 80%). All statistical analyses were conducted using the R 4.3.2 software (R Core Team, 2023).

## Results

### *In vitro* tests

**Figure 2:**
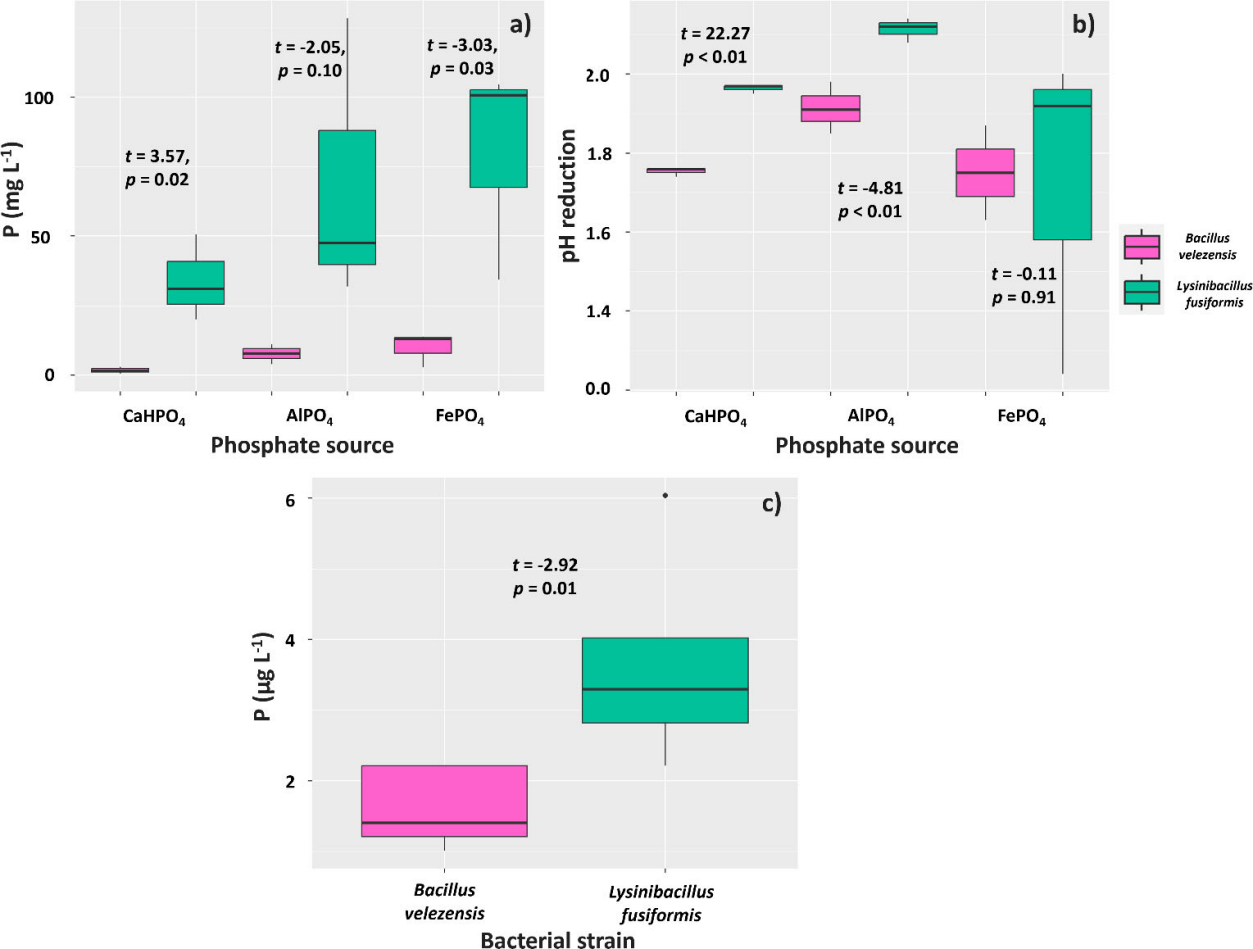
Solubilization of phosphate from CaHPO_4_, AlPO_4_ and FePO_4_ sources **(A)**, culture medium pH reduction **(B)**, and phosphate solubilization through phytase activity **(C)** by strains of *Bacillus velezensis* (BVPS01) and *Lysinibacillus fusiformis* (BVPS02). Data acquired 72 hours after incubation. Horizontal bars within boxplots represent the median of three replications per treatment. Values above the boxes were obtained using the Student’s t-test (p< 0.05).

The solubilization of phosphorus (P) supplied through CaHPO_4_ and FePO_4_ by the tested strains of *Bacillus velezensis* and *Lysinibacillus fusiformis* (BVPS01 and BVPS02, respectively) was different. The mean P solubilizations were 30.57 mg L^-1^ for *L. fusiformis* and 0.55 mg L^-1^ for *B. velezensis* (**Figure 2A**). Similarly, *L. fusiformis* solubilized a higher amount of P (69.30 mg L^-1^) when using FePO_4_ as a P source compared to *B. velezensis* (7.59 mg L^-1^). The pH of the growth media showed greater reductions for the metabolism of *L. fusiformis*, as this bacterium acidified the medium supplemented with CaHPO_4_ by 1.96 below that of the control, whereas *B. velezensis* acidified the medium by 1.75 (**Figure 2B**). Regarding the growth medium with AlPO_4_, *L. fusiformis* also acidified the medium more (2.11) than *B. velezensis* (1.91), although it did not result in higher P solubilization.

The phytase enzyme was not found in samples obtained from lysates of the BVPS01 and BVPS02 strains. However, evaluations of the supernatant showed a mean concentration of 3.60µg L^-1^ of P in the culture medium with *L. fusiformis* (BVPS02) and a lower concentration (1.61 µg L^-1^) in the medium with *B. velezensis* (**Figure 2C**).

**Figure 3:**
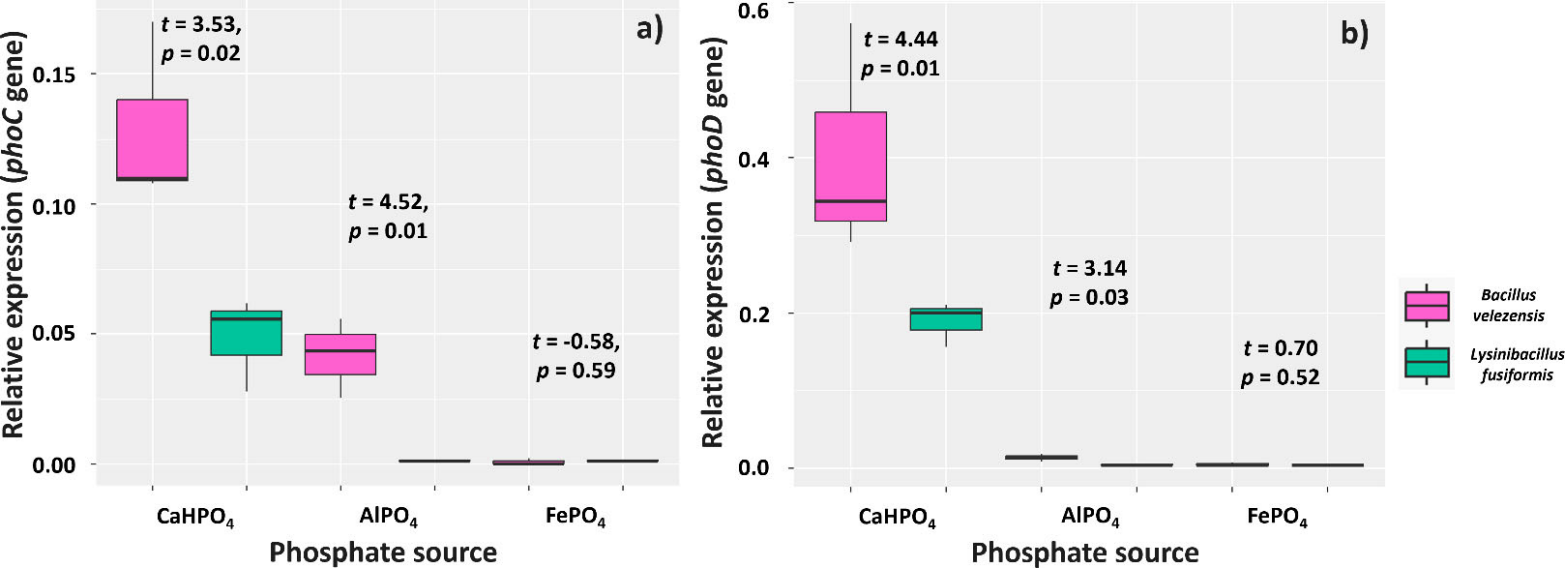
Relative expression of phoC ***(*A*)*** and phoD ***(*B*)*** genes in media containing CaHPO_4_, AlPO_4_, and FePO_4_ as phosphorus sources. Functional trait found for strains of *Bacillus velezensis* (BVPS01) and *Lysinibacillus fusiformis (*BVPS02). Data acquired 48 hours after incubation and normalized using the Eub gene as a reference. Horizontal bars within boxplots represent the median of three replications per treatment. Values above the boxes were obtained using the Student’s t-test (p< 0.05).

Overall, the BVPS01 and BVPS02 strains were more stimulated to express the phoC and phoD genes in the presence of CaHPO_4_ and reacted poorly to the other P sources, mainly regarding phoD (**Figures 3A, B**). Culture media with CaHPO_4_ and FePO_4_ sources and *B. velezensis* showed higher expression of phoC (0.13 and 0.04, respectively) than those with *L. fusiformis* (0.04 and 0.001, respectively). The strains also showed different expression of phoD for the CaHPO4 and FePO4 sources: 0.40 and 0.013 (*B. velezensis*) and 0.18 and 0.004 (*L. fusiformis*), respectively.

**Figure 4:**
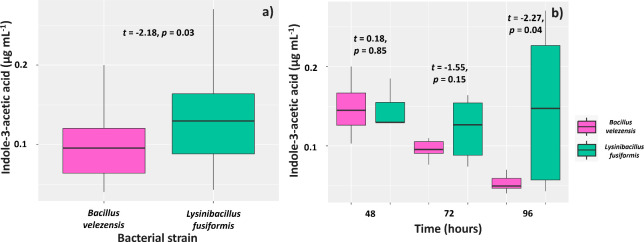
Synthesis of indole-3-acetic acid by strains of *Bacillus velezensis* (BVPS01) and *Lysinibacillus fusiformis* (BVPS02) **(A)** observed at 48, 72, and 96 hours of strain growth **(B)**. Horizontal bars within boxplots represent the median of three replications per treatment. Values above the boxes were obtained using the Student’s t-test (p< 0.05).

Overall, *L. fusiformis* produced significantly higher IAA levels (0.13 µg mL^-1^) than *B. velezensis* (0.09 µg mL^-1^) (**Figure 4A**). Assessments of IAA production in different culture times showed that the IAA levels found at the first evaluation (48 hours) in the culture media of the two strains were similar (0.10 and 0.09 µg mL^-1^, respectively). However, the IAA level in the culture medium of *L. fusiformis* was 0.08 µg mL^-1^ and lower in the culture medium with *B. velezensis* and *L. fusiformis* (0.06 µg mL^-1^) (**Figure 4B**) at 72 hours. Regarding the evaluation at 96 hours, IAA level was 0.09 µg mL^-1^ for *L. fusiformis*, but significantly lower for *B. velezensis* (0.03 µg mL^-1^).

**Figure 5:**
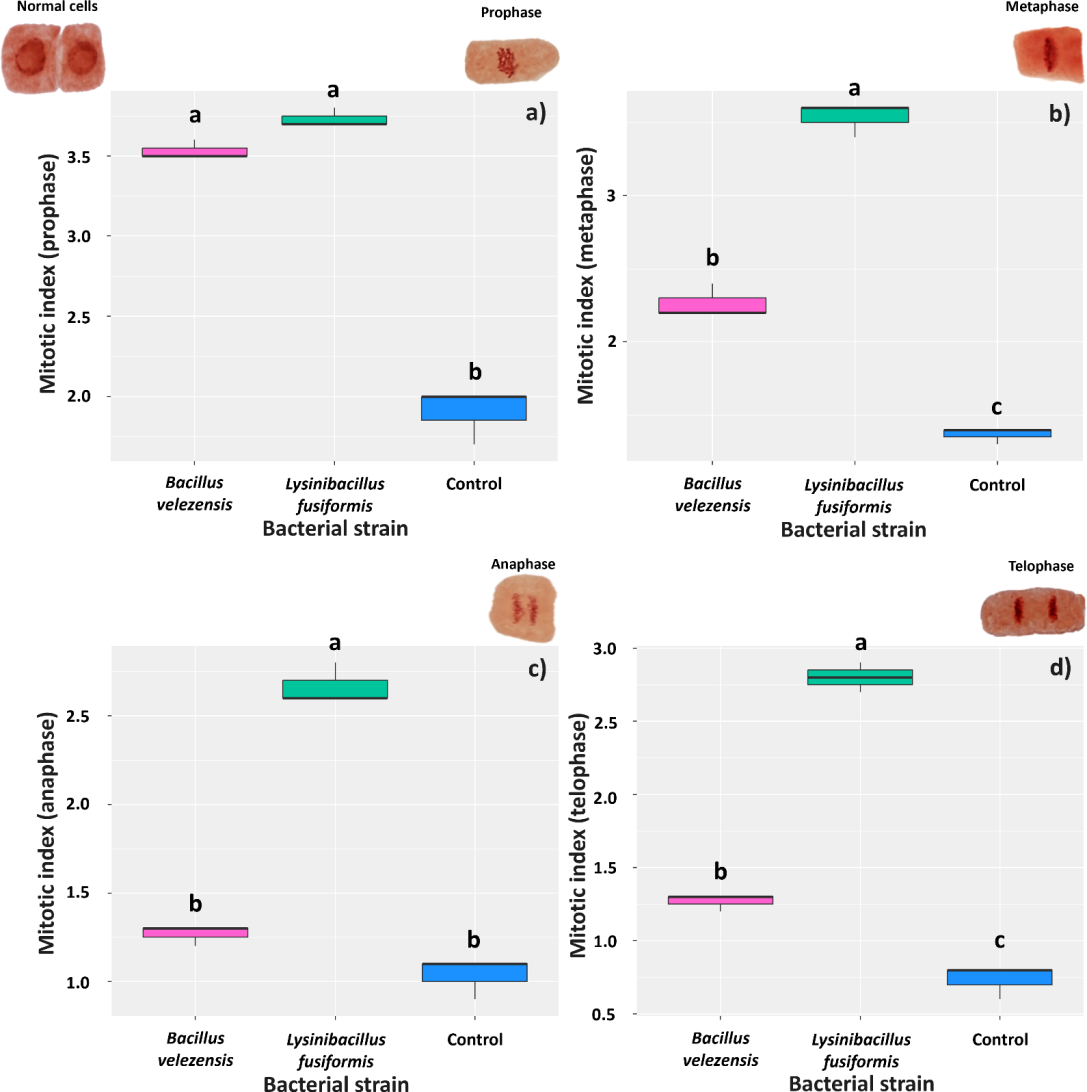
Mitotic index in meristematic cells of *Glycine max* roots at three days after inoculation with strains of *Bacillus velezensis* (BVPS01) and *Lysinibacillus fusiformis* (BVPS02). Index estimated for different phases of cell division: prophase **(A)**, metaphase **(B)**, anaphase **(C)**, and telophase **(D)**. Horizontal bars within boxplots represent the median of three replications per treatment (evaluation of 1,000 cells per replication). Boxes with equal letters are not significantly different by the Tukey’s test (p< 0.05). The images above the graphs are the different meristematic cells, representing the different phases of cell division.

The presence of the bacterial strains in the radicle stimulated cell division in the meristematic region at 3 days after inoculation. Comparison of mean number of cells in the prophase phase showed that soybean roots treated with *B. velezensis* or *L. fusiformis* showed mitotic indices of 3.53 and 3.73, respectively, whereas that found for control roots was 1.9 (**Figure 5A**). The bacteria also positively affected the mean number of cells in the metaphase phase; however, the highest mean number of cells dividing in this phase (3.53) was found for roots treated with *L. fusiformis* (3.53) (**Figure 5B**). This bacterium strain also increased the number of meristematic cells in the anaphase and telophase phases, with roots showing mitotic indices of 2.66 and 2.80, respectively (**Figures 5C, D**).

**Figure 6:**
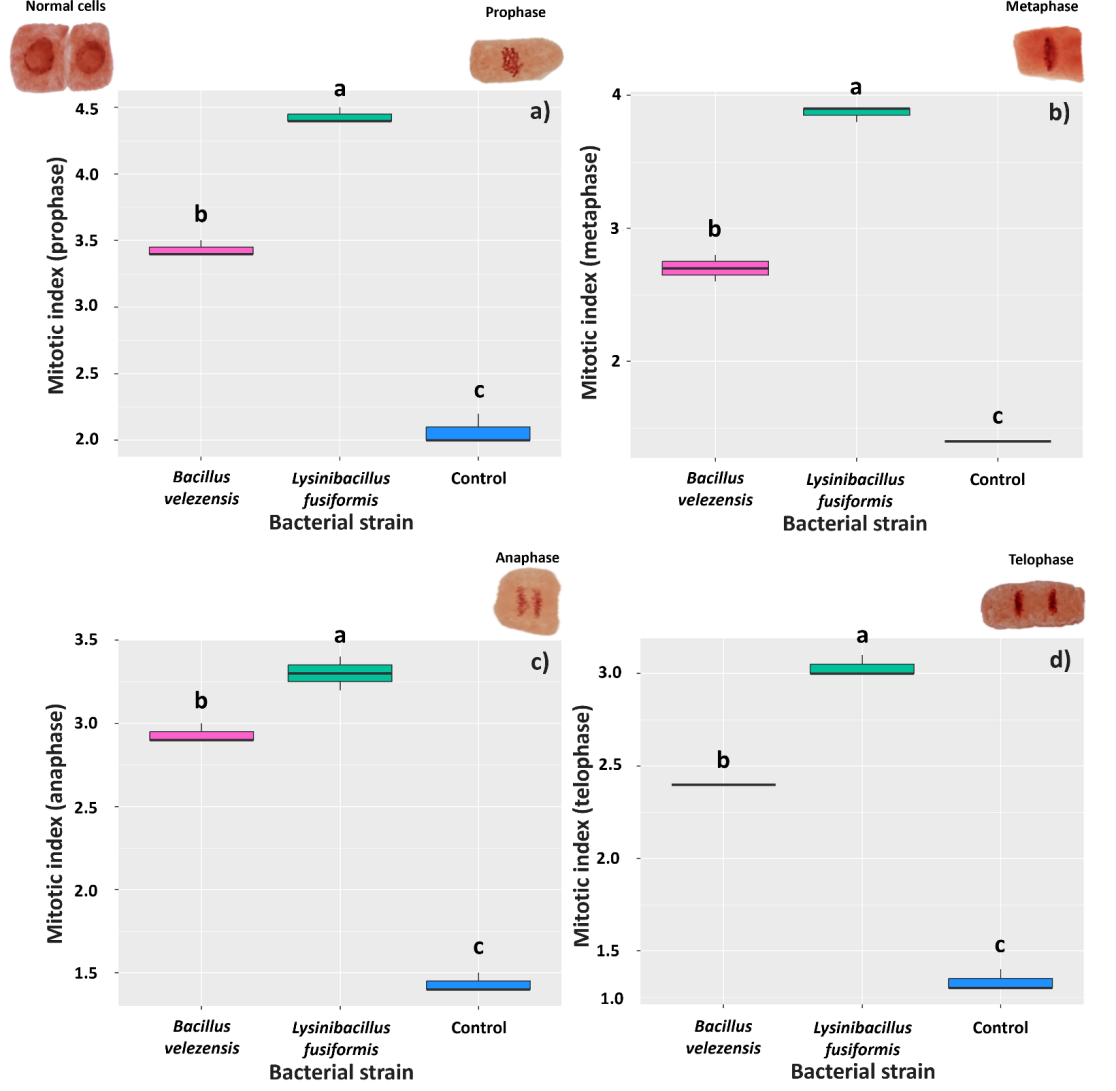
Mitotic index in meristematic cells of *Glycine max* roots at 6 days after inoculation with strains of *Bacillus velezensis* (BVPS01) and *Lysinibacillus fusiformis* (BVPS02). Index estimated for different phases of cell division: prophase **(A)**, metaphase **(B)**, anaphase **(C)**, and telophase **(D)**. Horizontal bars within boxplots represent the median of three replications per treatment (evaluation of 1,000 cells per replication). Boxes with equal letters are not significantly different by the Tukey’s test (p< 0.05). The images above the graphs are the different meristematic cells, representing the different phases of cell division.

Root responses to the presence of the bacteria at 6 days after inoculation were similar in all the evaluated mitotic phases. Roots treated with *L. fusiformis* showed the highest mean indices, regardless of the mitotic phase: 4.43 (prophase), 3.86 (metaphase), 3.30 (anaphase), and 3.03 (telophase); the mean mitotic indices found for the control roots were 2.06, 1.40, 1.43, and 1.33, respectively (**Figures 6A–D**).

**Figure 7:**
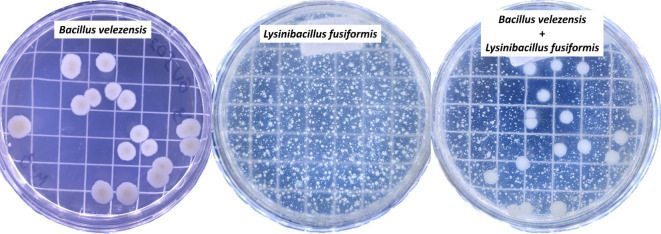
Biological compatibility between the strains BVPS01 (*Bacillus velezensis*) and BVPS02 (*Lysinibacillus fusiformis*). Individual plating of BVPS01, BVPS02, and plating of the biofertilizer consisting of the mixture of the two rhizobacteria (*Bacillus velezensis* + *Lysinibacillus fusiformis*).

The evaluated rhizobacterial strains exhibited a pattern of biological compatibility, showing no antibiotic halos, colonial pattern modification, or significant reduction in colony number (**Figure 7**). *B. velezensis* developed a mean of 1.9 × 10^8^ CFU mL^-1^ when grown alone and 2.0 × 10^8^ CFU mL^-1^ when grown together with *L. fusiformis*. However, a mean of 1.6 × 10^11^ CFU mL^-1^ was found for *L. fusiformis* when grown alone and 1.4 × 10^11^ CFU mL^-1^ when grown in together with *B. velezensis*.

### Field tests

**Figure 8:**
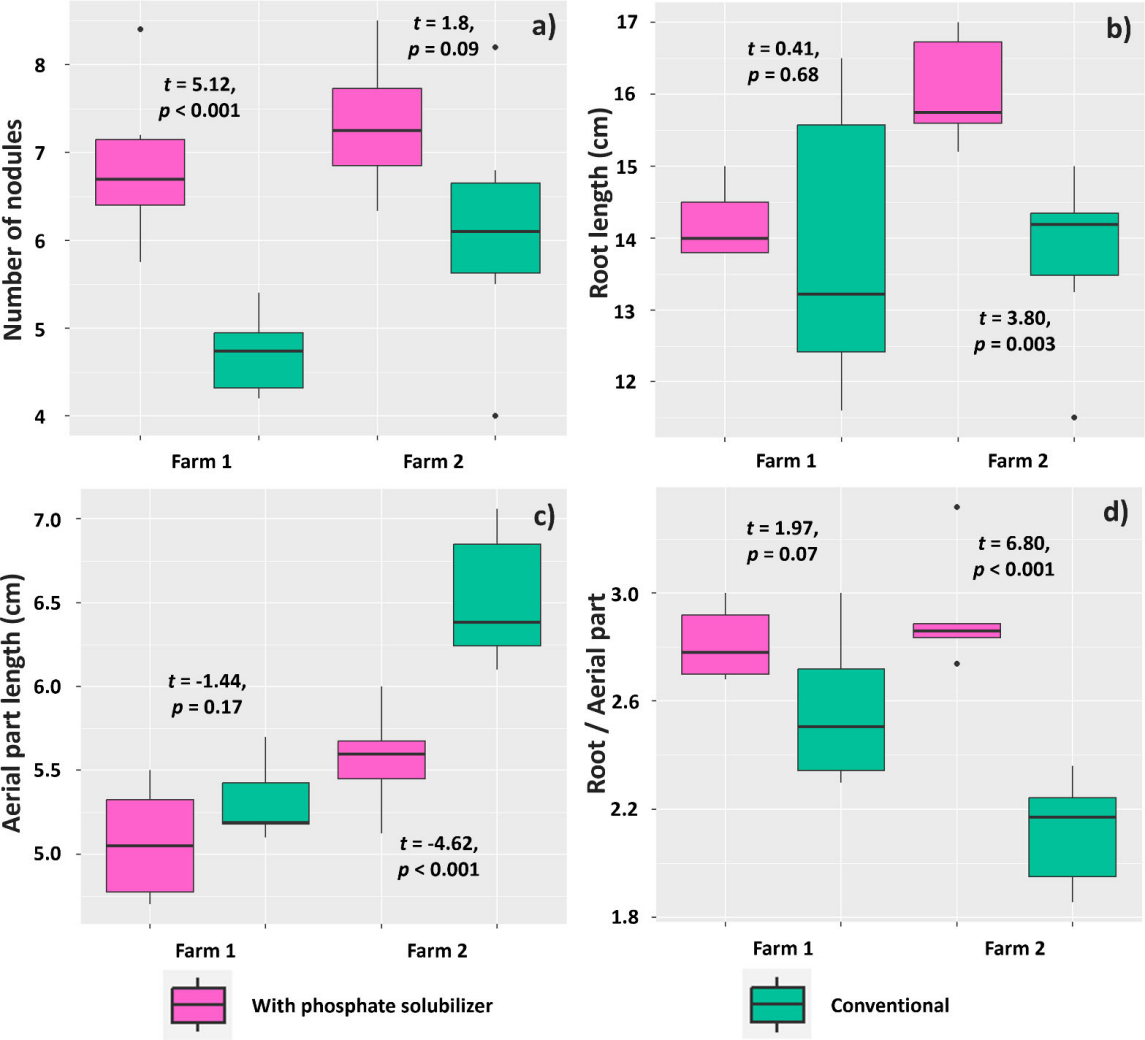
Number of nodules **(A)**, taproot length **(B)**, shoot length **(C)**, and root-to-shoot ratio **(D)** in *Glycine max* seedlings subjected to or not subjected to (conventional treatment) inoculation with a biofertilizer based on phosphate-solubilizing rhizobacteria (*Bacillus velezensis* and *Lysinibacillus fusiformis*).

The mean number of nodules on soybean plants was affected by the treatment with biofertilizer based on phosphate-solubilizing rhizobacteria (PSR) only in the experiment in Farm 1, where plants inoculated with PSR showed a mean of 6.85 nodules per plant, whereas plants subjected to the conventional treatment showed a mean of 4.71 (**Figure 8A**). However, taproot length was affected by the treatments only in the experiment in Farm 2, where plants subjected to the PSR-based biofertilizer had a longer taproot (16.05 cm) than those without the biofertilizer (13.75 cm) (**Figure 8B**). Mean shoot length was also affected by inoculation with *B. velezensis* and *L. fusiformis* in Farm 2; however, plants inoculated with these rhizobacteria invested less in above-ground biomass (5.57 cm) than those under the conventional treatment (6.52 cm) (**Figure 8C**). Consequently, the root-to-shoot ratio was affected by inoculation with these rhizobacteria in Farm 2, as plants subjected to this treatment showed a higher root-to-shoot ratio (2.91) than those subjected to the conventional treatment (2.11) (**Figure 8D**).

**Figure 9:**
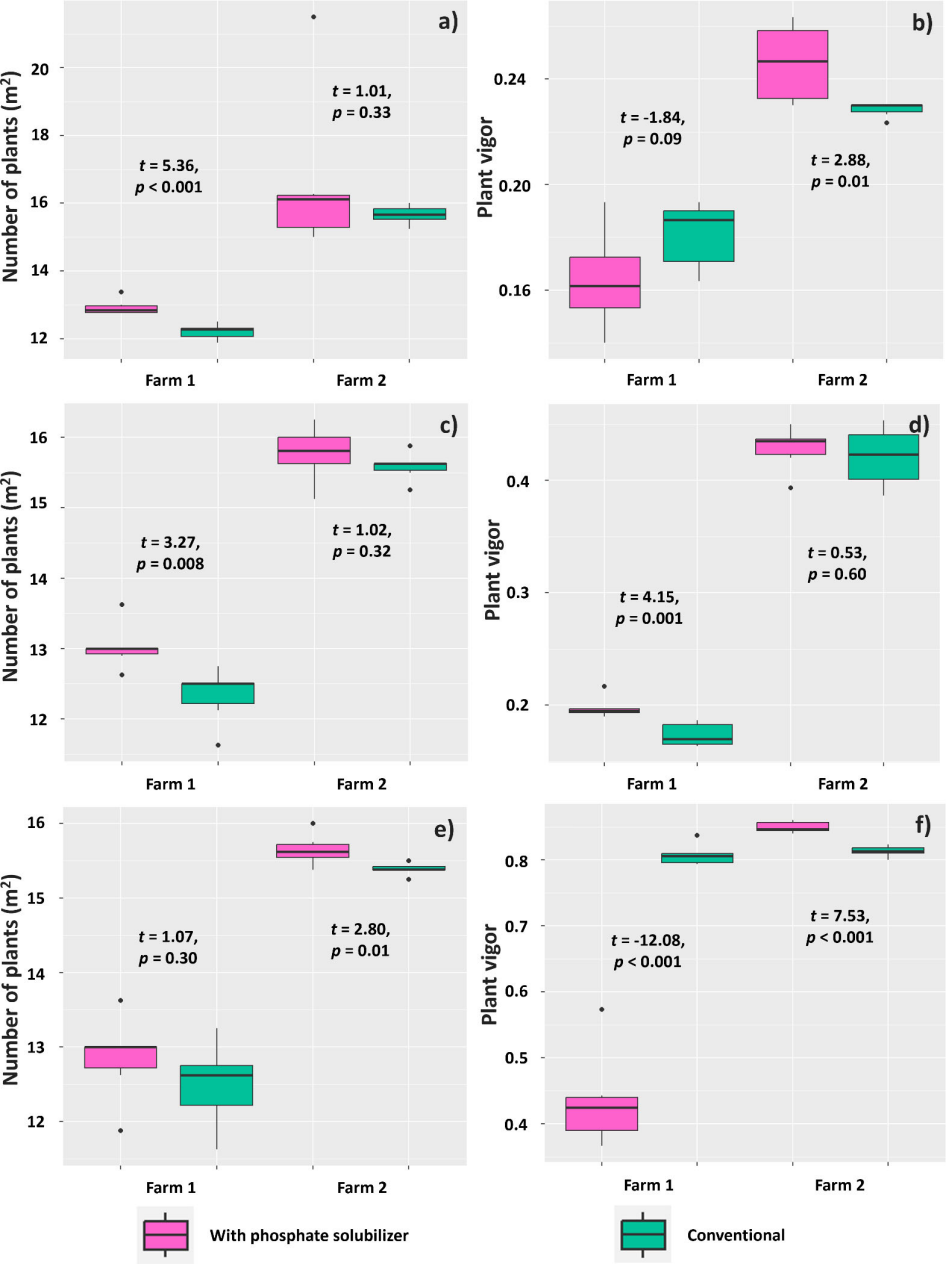
Mean number of plants per m^2^ and plant vigor assessed at 14 **(A, B)**, 21 **(C, D)**, and 35 **(E, F)** days after emergence of *Glycine max* plants subjected or not (conventional treatment) to inoculation with a biofertilizer based on phosphate-solubilizing rhizobacteria (*Bacillus velezensis* and *Lysinibacillus fusiformis*). Data acquired in experiments conducted in 2 farms. Horizontal bars within boxplots represent the median of six replications per treatment. Points outside the boxes are outliers. Values above the boxes were obtained using the Student’s t-test (p< 0.05).

Evaluation at 14 DAE showed effects of using the PSR-based biofertilizer on the number of established plants only in Farm 1, resulting in a higher mean plant density per m^2^ (12.92) than the treatment without the biofertilizer (12.20) (**Figure 9A**). The vigor of plants in Farm 2, however, was affected by using the biofertilizer, which resulted in a mean vigor (0.25) higher than that found for plants in the conventional treatment (0.22) (**Figure 9B**). Regarding the evaluation at 21 DAE, the number of plants in Farm 1 presented similar results, with a difference between plants under biofertilizer treatment (13.05 plants m^2^) and those under conventional treatment (12.33 plants m^2^) (**Figure 9C**). Plant vigor was also higher (0.19) in the biofertilizer treatment than that in the conventional treatment (0.17) (**Figure 9D**).

Evaluation at 35 DAE showed that the treatments affected the mean number of plants per m^2^ only in Farm 2, where plants treated with the biofertilizer had a higher mean (15.64) than those without the biofertilizer (15.38) (**Figure 9E**). Plant vigor at this period showed an effect of treatments on both farms: plants in Farm 1 had a higher mean vigor (0.80) when subjected to the conventional treatment, whereas those in Farm 2 had higher vigor (0.84) when subjected to the treatment with the biofertilizer based on phosphate-solubilizing rhizobacteria (**Figure 9F**).

**Figure 10:**
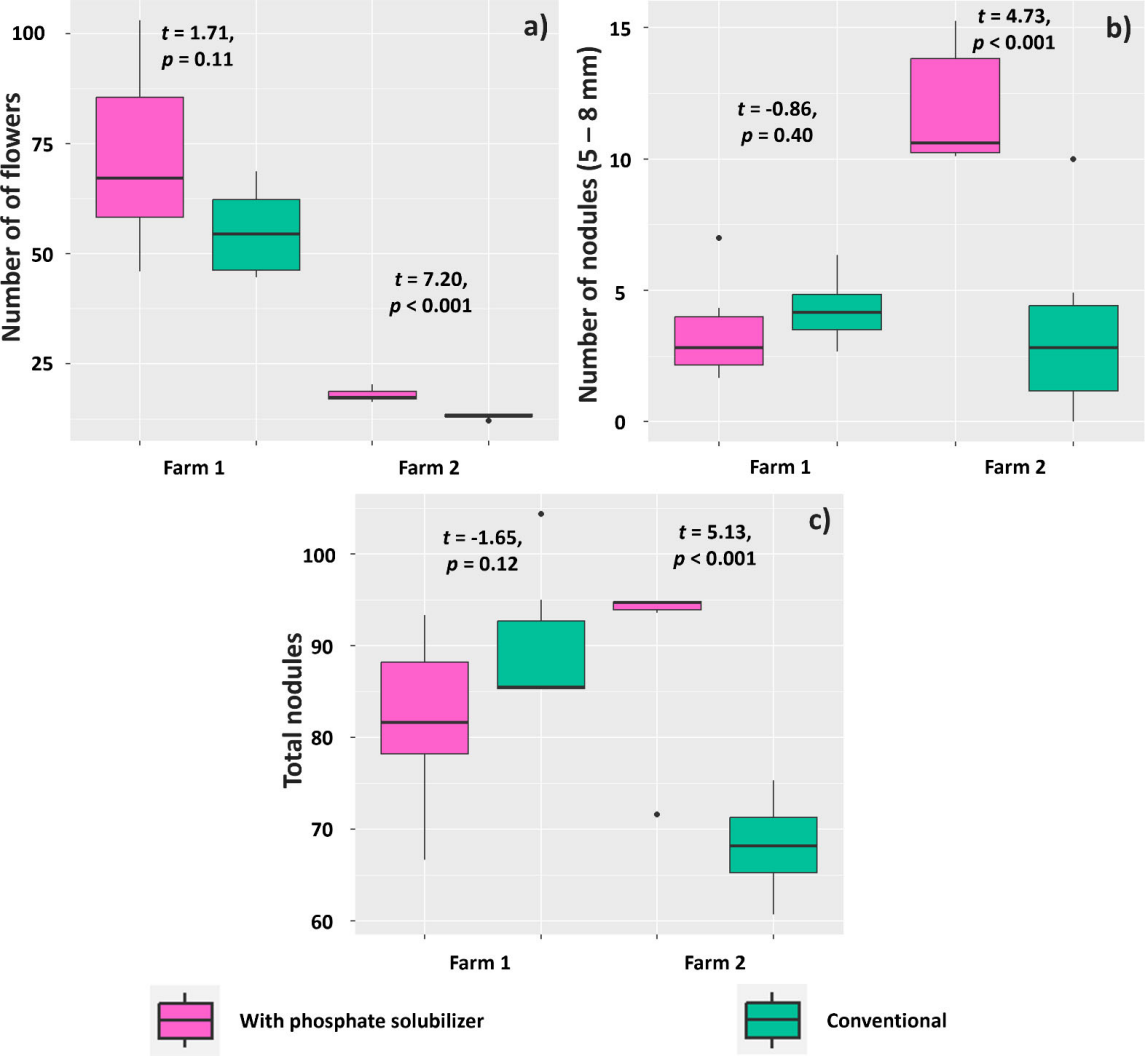
Mean number of flowers **(A)**, nodules of 5–8 mm in diameter **(B)**, and total number of nodules **(C)** in *Glycine max* plants subjected or not (conventional treatment) to inoculation of a biofertilizer based on phosphate-solubilizing rhizobacteria (*Bacillus velezensis* and *Lysinibacillus fusiformis*). Data acquired at 35 days after emergence in experiments conducted in 2 farms. Horizontal bars within boxplots represent the median of six replications per treatment. Points outside the boxes are outliers. Values above the boxes were obtained using the Student’s t-test (p< 0.05).

Regarding the experiment in Farm 1, the treatment with PSR-based biofertilizer affected the number of flowers, number of nodules (diameter of 5–8 mm), and total number of nodules evaluated at 35 DAE. Plants in this treatment had a mean of 17.88 flowers, whereas those in the conventional treatment had 13.11 flowers (**Figure 10A**). The number of large nodules (diameter between 5 and 8 mm) was also higher in plants treated with the biofertilizer (11.93) compared to those in the conventional treatment (3.53) (**Figure 10B**). The total number of nodules, regardless of diameter, was also higher in plants subjected to the biofertilizer treatment (90.71) compared to that found for plants without this treatment (60.15) (**Figure 10C**).

**Figure 11:**
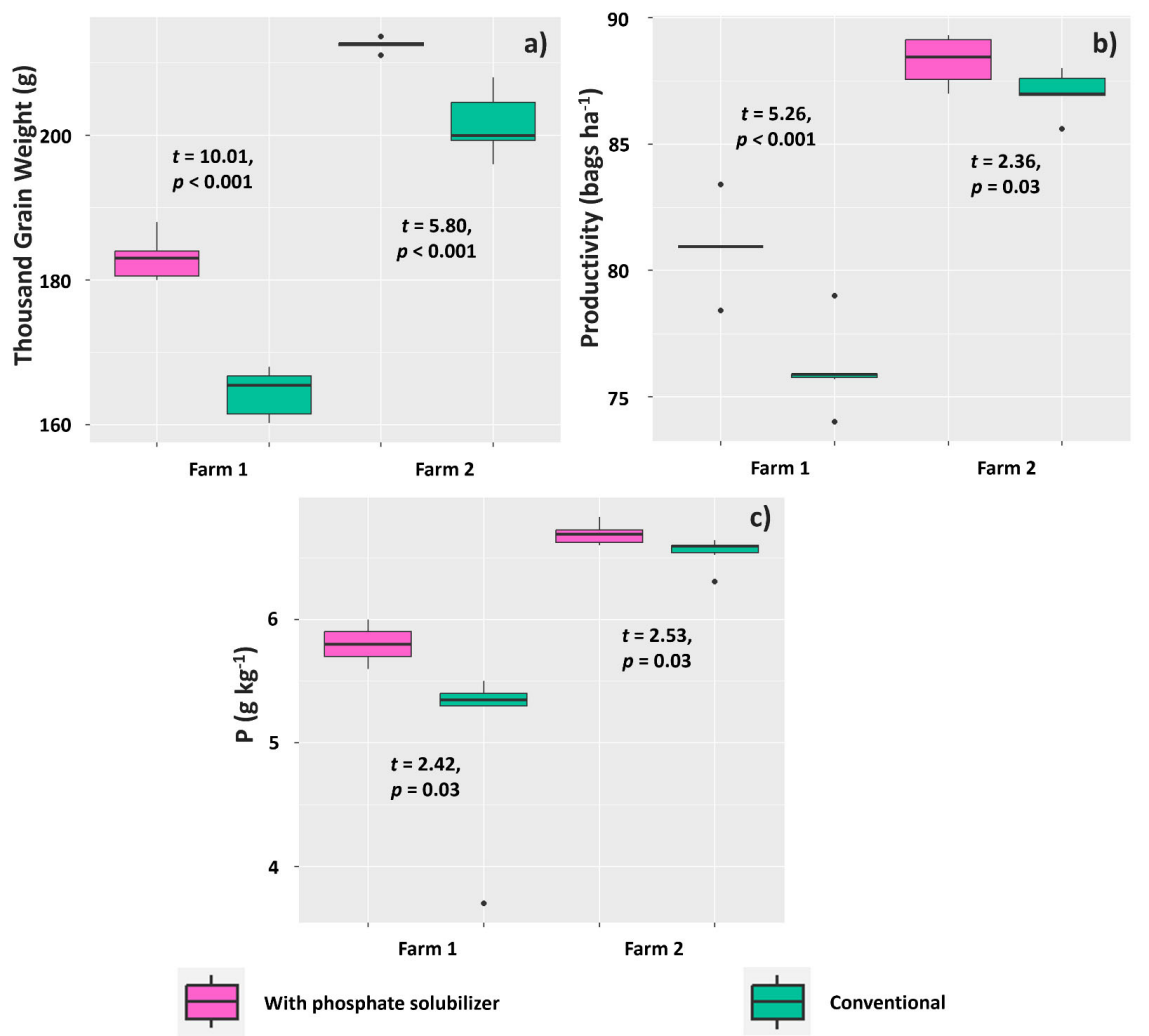
Mean 1000-grain weight **(A)**, grain yield **(B)**, and P content **(C)** in grains in Glycine max plants subjected to or not subjected to (treatment conventional) inoculation of a biofertilizer based on phosphate-solubilizing rhizobacteria (*Bacillus velezensis* and *Lysinibacillus fusiformis*).

The treatments affected 1000-grain weight, grain yield, and P content in grains of soybean plants evaluated on both farms. The means found for these variables were higher for plants treated with the PSR-based biofertilizer on both farms. The mean 1,000-grain weights were 183 and 164.41 g (Farm 1) and 212.46 and 201.50 g (Farm 2) for plants subjected to the biofertilizer and conventional treatments, respectively (**Figure 11A**).

Regarding grain yield, the farms presented a similar trend; the averages were 80.93 and 76.06 60-kg bags ha^-1^ (Farm 1) and 88.30 and 87.05 60-kg bags ha^-1^ (Farm 2) for plants subjected to the biofertilizer and conventional treatments, respectively (**Figure 11B**). Soybean grains from plants treated with the PSR-based biofertilizer tended to accumulate more P than those under the conventional treatment: the means found were 5.8 and 5.1 g kg^-1^ (Farm 1) and 6.7 and 6.5 g kg^-1^ (Farm 2) for the biofertilizer and conventional treatments, respectively (**Figure 11C**).

**Figure 12:**
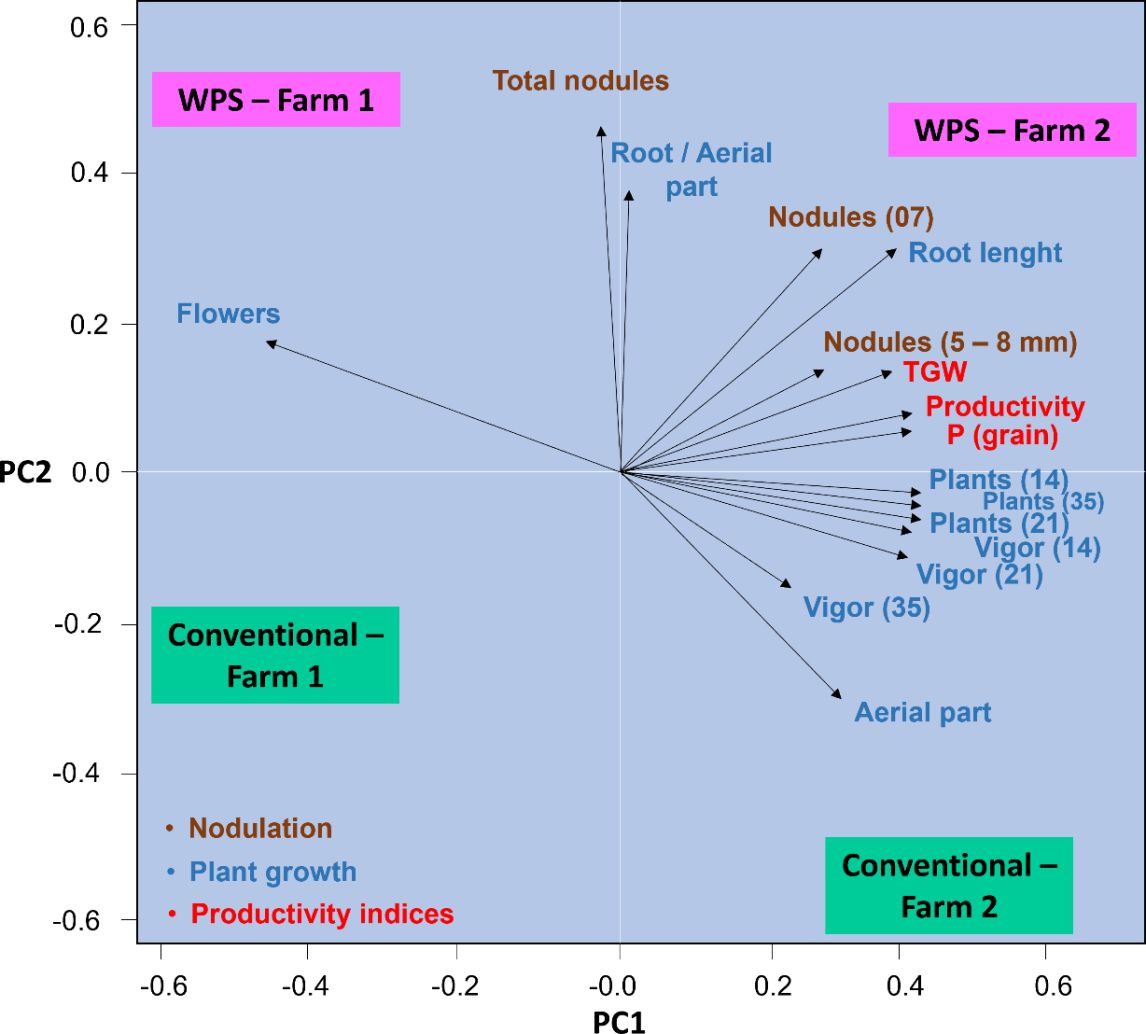
Principal component analysis for plant growth, nodulation, and yield data for *Glycine max* subjected or not (treatment conventional) to inoculation with a biofertilizer based on phosphate-solubilizing rhizobacteria (*Bacillus velezensis* and *Lysinibacillus fusiformis*). Data acquired in experiments conducted in 2 farms. WPS = phosphate-solubilizing rhizobacteria; TGW = 1000-gain weight. Numbers in the variables indicate the evaluation period (7, 14, 21, or 35 days after emergence).

Principal component analysis (PCA) showed that components 1 and 2 together explained 93% of the data variance. The results denoted that the use of a PSR-based biofertilizer for growing soybeans in Farm 2 explained the most variance in data of root-to-shoot ratio and taproot length, which had the highest means (**Figure 12**). Additionally, the highest means for nodulation and yield-related variables were found in the treatment containing the biofertilizer. The use of the conventional treatment in Farm 1, however, resulted in the lowest means for all analyzed variables. Inoculation with PSR resulted in soybean plants investing more in flowers in Farm 1, while the conventional treatment resulted in plants investing more in shoots in Farm 2, which, although it did not result in higher crop yield.

## Discussion

### The *Lysinibacillus fusiformis* strain BVPS02 can solubilize phosphates through organic acid- and phytase-related pathways, as well as can synthesize IAA and improve mitotic index in the root meristem of soybean plants

Although *L. fusiformis* has been little explored as a plant growth-promoting bacterium, the genus *Lysinibacillus* hosts bacteria such as *L. sphaericus*, *L. pakistanensis*, *L. varians*, and *L. macroides* that have well-documented potential for phosphate solubilization, indole-3-acetic acid (IAA) synthesis, and biocontrol of plant pathogens (Naureen et al., 2017; Pal and Sengupta, 2019; Jyolsna et al., 2021; Lelapalli et al., 2021; Hernández-Santana et al., 2022). Pantoja-Guerra et al. (2023) reported that six different strains of *Lysinibacillus* spp. Increased biomass production and modified root architecture in maize plants, which may be attributed to the stimulation of genes involved in the IAA biosynthesis. Additionally, they found that culture filtrates from these six strains increased maize coleoptile length, similarly to IAA, denoting that the filtrates had a similar effect to auxin on plant tissue. Recently, Jha and Mohamed (2023) inoculated maize plants with an *L. fusiformis* strain that can solubilize P and produce gluconic acid, phytohormones, siderophores, and catechol. They found that the strain positively regulated osmolytes, phenolics, phytohormones, and antioxidant enzymes in the plants, inducing resistance to osmotic and oxidative stresses caused by exposure to cold.

*L. fusiformis* effectively reduced the pH of media containing CaHPO_4_ and FePO_4_ as P sources, indicating solubilization through organic acid production. These acids exuded by some phosphate-solubilizing rhizobacteria (PSR) (Marra et al., 2019) can chelate cations bound to phosphate through their hydroxyl and carboxyl groups, converting it into soluble forms (Mardad et al., 2013). They can also reduce rhizosphere pH through gas exchange (O_2_/CO_2_) and proton-bicarbonate balance, thus releasing associated P (Kim et al., 1997). Organic acids, including citric, gluconic, oxalic, and tartaric acids, not only release P bound to calcium, iron, and aluminum oxides but are also responsible for solubilizing tricalcium and rock phosphates (Prijambada et al., 2009; Kishore et al., 2015). Fermentation, respiration of organic carbon compounds, and direct oxidation are important metabolic pathways for organic acid synthesis by PSR, resulting in acidification around cells and release of phosphate complexes by proton substitution for cations such as Ca^+2^, Fe^+3^, and Al^+3^ or by phosphate (PO_4_^2-^) exchange for acidic anions (Rawat et al., 2021).

*L. fusiformis* exhibited not only phosphate solubilization activity but also participated in mineralization processes, releasing P from organic sources through the synthesis of phytate-type phosphatases. The plant potential to obtain P from phytate is significantly limited. Thus, using mineralizing bacteria that produce phytate for growing cereal crops has resulted in increased P uptake without the need for fertilizer use (Martínez et al., 2015). Moreover, the introduction of phytases into the soil through the activity of PSR such as *L. fusiformis* can improve plant growth and increase crop yield, making it a sustainable and low-cost strategy (Singh et al., 2020).

The results also showed that *L. fusiformis* can increase the mitotic index in the meristematic region of *G. max* roots. Studies have documented that exopolysaccharides released by plant growth-promoting rhizobacteria can activate cell divisions in root meristems. Tkachenko et al. (2015) reported an increase in mitotic activity of meristematic cells in roots of potato microclones due to inoculation with *Azospirillum*, improving plant yield and adaptability. Inoculation with *Paenibacillus polymyxa* increased the mitotic index in the root meristem of wheat plants by 2.8 times, resulting in a 22% increase in root size (Yegorenkova et al., 2021). Additionally, lipopolysaccharides that participate in the cell wall composition of PSR increase the mitotic index in root meristematic cells, determining the interactions of bacteria and roots and participating in the induction of plant responses to these interactions (Evseeva et al., 2011). According to Shirokov et al. (2020), even the glycosylated flagellin from the polar flagellum of the plant growth-promoting rhizobacterium *Azospirillum brasilense* positively increased the division rate of meristematic cells in wheat roots, denoting elicitor properties of rhizobacterial flagella. The cell division-inducing effects of *L. fusiformis* explain the root growth found in *G. max* plants inoculated with the PSR-based biofertilizer and grown in the field. This highlights the need for technological exploitation of *L. fusiformis*, emphasizing Titânico^®^, which is currently the only known registered commercial product based on strains of this bacterium.

### The *Bacillus velezensis* strain BVPS01 shows increased expression of the phoC and phoD genes in the presence of the CaHPO_4_ complex, indicating its potential for solubilization through phosphatase activity

Different *B. velezensi*s strains have been described in the literature as potential plant growth-promoting bacteria for important agricultural crops, including sugarcane, wheat, maize, and soybean. Thus, this bacterium has been considered a new multifunctional agent for phosphate solubilization, nutrient uptake improvement, and biocontrol (e.g., Meng et al., 2016; Mosela et al., 2022; Santos et al., 2022; Wang et al., 2022; Afzal et al., 2023). Bayisa (2023) reported that *B. velezensis* can efficiently solubilize tricalcium phosphate (Ca-P) and aluminum phosphate (Al-P), producing significant amounts of succinic and 2-ketogluconic acids (in medium supplemented with Ca-P) and 2-ketogluconic and fumaric acids (in medium supplemented with Al-P), as well as significant extracellular acid and alkaline phosphatase activities.

The expression of the phoC and phoD genes was activated in the tested *B. velezensis* strain (BVPS01), mainly the presence of CaHPO_4_ as a P source. These genes encode for non-specific acidic (phoC) and alkaline (phoD) phosphatases (Fraser et al., 2017; Luo et al., 2019) in soil microorganisms. Alkaline phosphatases control the hydrolysis of phosphomonoesters and phosphodiesters (Wang et al., 2021), but both acidic and alkaline phosphatase activities have been used as proxies to assess the mineralization of organic P into bioavailable inorganic P (Luo et al., 2019). Zheng et al. (2022) analyzed the abundance of expression of the functional phoC and phoD genes of PSR in maize rhizosphere and non-rhizosphere areas and found that phosphatase activity and the number of gene copies of phoC and phoD were higher in rhizosphere soils than in non-rhizosphere soils. Thus, PSR-based bioproducts that have at least one active copy of phosphatase production-related genes in their genomes may contribute to increases in phosphatase activity in the soil (Wan et al., 2020), constituting an important mechanism for improving P uptake by roots.

According to Dai et al. (2020), phosphatase release under low-P conditions could be increased by regulating a gene (phoR) involved in regulating the response to phosphorus deficiency to mineralize soil organophosphates. Pan and Cai (2023) reported that alkaline phosphatase is more secreted and, therefore, genes encoding this enzyme may be more common in PSR. The results of the present study confirmed that *B. velezensis* can express phoD more effectively than phoC; however, this expression occurred even under high-P conditions, indicating an important pathway for P acquisition by this bacterium, even under high P concentrations. Additionally, this mineralization pathway appears to be more effective for *B. velezensis* to obtain P than the solubilization pathway.

Considering the well-documented potential of *B. velezensis* in the literature, this rhizobacterium is recognized as a model plant growth-promoting bacterium for its outstanding performance in improving the growth and protection of many species of cultivated plants. Consequently, various commercial products based on strains of this bacterium have stood out in the current market of bioinputs (Jang et al., 2023; Wang, 2023). Therefore, the results of the present study allow for the association of this phosphate-solubilizing bacterium with a bacterium that has been little evaluated for plant growth promotion, resulting in the composition of a commercial product that improved the performance of *G. max* plants in the field.

### *B. velezensis* and *L. fusiformis* are biologically compatible and, therefore, can be used to formulate a plant growth-promoting product

Biological compatibility among microorganisms is an essential factor to consider when developing commercial formulations containing two or more microbial strains. The literature has provided consistent information about antibiotic interactions between biocontrol rhizobacteria and plant pathogens (e.g., Liu et al., 2020; Rabbee et al., 2023). However, the development of commercial formulations and the combined application of biocontrol agents should account for aspects of compatibility between microorganisms. Rhizobacteria can be mechanically incompatible, as one strain interferes with the mechanism by which a second strain acts (Stockwell et al., 2011). Anderson et al. (2004) found an antagonistic effect between biocontrol bacterial strains (*Pseudomonas fluorescens* A506 and *Pantoea agglomerans* Eh252); an extracellular protease produced by strain A506 inactivates antibiotics produced by strain Eh252, affecting its functional trait. Thus, mixtures of PSR may perform better in providing P to plants than compositions of single strains of P solubilization or mineralization due to synergistic effects, resulting in greater efficacy in the field, provided that they do not exhibit antibiosis.

### The *B. velezensis* and *L. fusiformis*-based biofertilizer showed potential to increase root development, number of nodules and flowers, and consequently 1000-grain weight, grain yield, and P content in soybean grains

Plant growth-promoting rhizobacteria can affect plant root architecture, vegetative growth, and physiology. IAA production is directly connected to changes in the root system (Turan et al., 2021), as IAA stimulates root cell proliferation, increasing the mitotic index in roots and consequently the number of lateral roots and root hairs (Takahashi, 2013; Guan et al., 2019). Several rhizobacteria affect the auxin-to-cytokinin ratio and produce secondary metabolites that interfere with the auxin synthesis pathway, elongating primary roots (Olatunji et al., 2017). IAA can be produced plant growth-promoting rhizobacteria through two metabolic pathways (indole-3-acetamide and indole-3-pyruvate), which are affected by the activity of the enzyme nitrate reductase, which can generate nitric oxide during root colonization (Sun et al., 2015). Nitric oxide is involved in the auxin signaling system, controlling lateral root development (Molina-Favero et al., 2007; Ma et al., 2020). The functional trait of *L. fusiformis* for IAA production explains its positive effect on root meristem cell division and root development in soybean plants inoculated with Titânico^®^ in the present study.

Research studies have shown that IAA is also involved in cell division, differentiation, and formation of vascular bundles in nodules (Mathesius, 2008; Gao et al., 2021). Root nodules seem to contain more IAA than non-nodulated roots, and IAA may be essential for maintaining root nodules (Kohlen et al., 2018). Nod factors initiate multiple essential responses for root colonization by nitrogen-fixing bacteria, such as those of the genera *Rhizobium* and *Bradyrhizobium*. One of the plant’s initial responses to Nod factor is an increase in intracellular calcium contents in root hairs, followed by significant calcium oscillations (spiking) and alterations in the root hair cytoskeleton. These responses are followed by curling of root hairs to trap the bacteria. Simultaneously, the Nod factor stimulates cortical root cells to reinitiate mitosis, which is a process dependent on auxin transport inhibition. These cells form the nodule primordium and give rise to cells that will receive the invading bacteria (Ghosh et al., 2011). Thus, products based on phosphate-solubilizing rhizobacteria that synthesize IAA can affect the pattern of root colonization by nitrogen-fixing bacteria and, therefore, the number and activity of nodules. Studies have shown that the co-inoculation of IAA-producing *Bacillus* bacteria and *Bradyrhizobium* bacteria can positively affect the growth and yield of agricultural crops (Atieno et al., 2012). Additionally, the combination of *Bacillus* and *Bradyrhizobium* bacteria positively affects P acquisition by plants (Akhtar et al., 2013; Korir et al., 2017; Matse et al., 2020).

Therefore, the increase found in for 1000-grain weight, grain yield, and P content in soybean plants subjected to application of a biofertilizer composed of phosphate-solubilizing rhizobacteria can be attributed not only to the effects of these rhizobacteria on phosphate solubilization and mineralization, but also to their effect on root architecture. Thus, the findings of the present study confirm the agricultural effectiveness of the *Bacillus velezensis* and *Lysinibacillus fusiformis*-based biofertilizer. Furthermore, current agriculture demands sustainable practices that simultaneously improve the value of grains, including increasing in essential nutrient contents such as N and P. Therefore, the bacterial strains tested in this study deserve further studies to expand the information about plant-microorganism interaction mechanisms and contribute to the development of strategies to increase the nutritional quality of soybean grains.

## Conclusions

The results of the study confirmed the hypothesis that combining the well-established phosphate-solubilizing bacterium *Bacillus velezensis* with the underexplored plant growth-promoting bacterium *Lysinibacillus fusiformis* can be used to formulate a biofertilizer that improves the performance of soybean plants in the field.

The *B. velezensis* strain (BVPS01) exhibited a functional trait for producing phosphatase enzymes by the expression of the phoC and phoD genes. The *L. fusiformis* strain (BVPS02) solubilized phosphates through organic acid- and phytase-related pathways, synthesized indole-3-acetic acid, and increased the mitotic index in the root meristem of soybean plants. These rhizobacterial strains showed biological compatibility.

The biofertilizer formulated based on these rhizobacteria increased root development, number of nodules and flowers, and positively affected 1000-grain weight, grain yield, and phosphorus content in grains. Thus, this biofertilizer showed potential to improve root growth and increase grain yield and quality of soybean crops, constituting a sustainable and low-cost strategy.

## Data Availability

The datasets presented in this study can be found in online repositories. The names of the repository/repositories and accession number(s) can be found in the article/**Supplementary Material**.
