# Supplementary material for: Effect of a Bacillus velezensis and Lysinibacillus fusiformis-based biofertilizer on phosphorus acquisition and grain yield of soybean
Source: Front Plant Sci. 2024 Aug 23;15:1433828. doi: 10.3389/fpls.2024.1433828 (PMC11378753; doi:10.3389/fpls.2024.1433828)
Supplement: Supplementary file 1 [file DataSheet1.pdf]

SUPPLEMENTARY MATERIAL

Effect of a *Bacillus velezensis* and *Lysinibacillus fusiformis*-based biofertilizer on phosphorus acquisition and grain yield of soybean

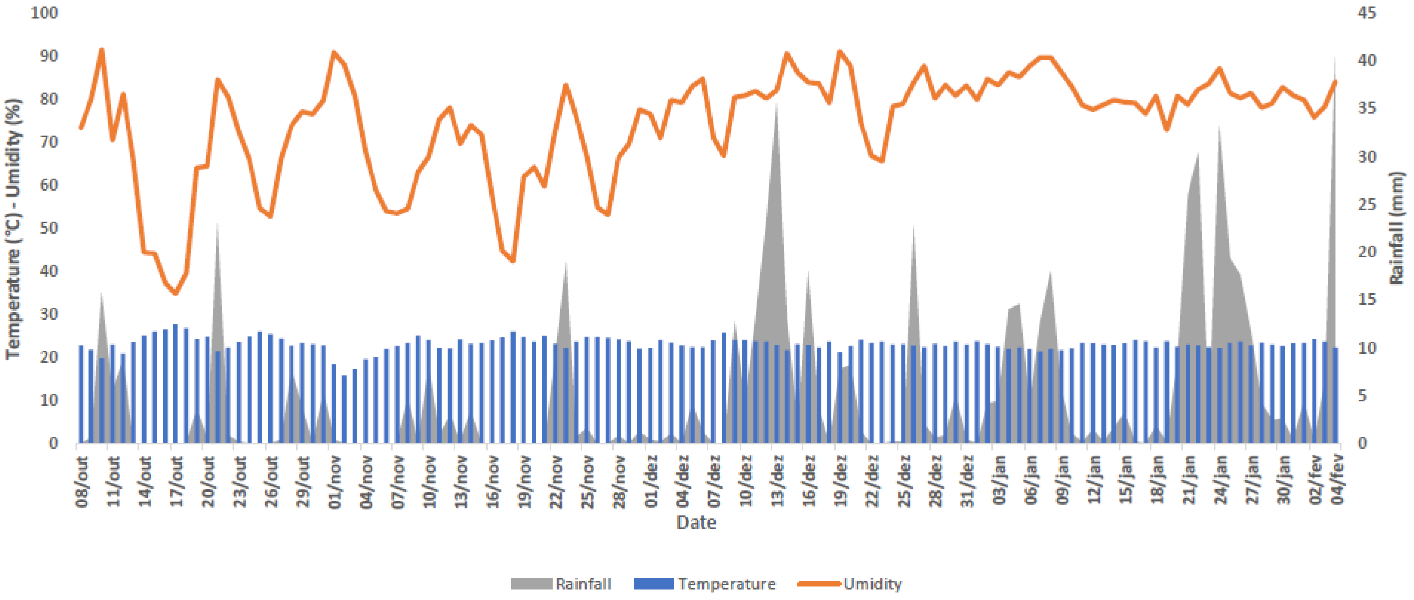

Figure S1. Climate data of temperature, relative air humidity, and rainfall depth during the experimental period (October 8, 2022 to February 4, 2023) for the southwest region of Goiás, Brazil, where experiments were conducted to evaluate the effect of using a biofertilizer based on phosphate-solubilizing rhizobacteria (*Bacillus velezensis* and *Lysinibacillus fusiformis*) on plant growth promotion in *Glycine max* crops.

Table S1. Description of the primers used for quantifying the expression of the phoC and phoD genes by rhizobacteria (*Bacillus velezensis* and *Lysinibacillus fusiformis*).

Target	Forward and Reverse Primers	Cycling parameters	Fragment	References
Eub	CCTACGGGAGGCAGCAG<br>ATTACCGCGGCTGCTGG	95°C - 10 min;<br>40 ciclos:<br>95°C - 30 s;<br>53°C - 40 s;<br>72°C - 40 s*<br>Melting	180 bp	Muyzer, et al., 1993
PhoC	CGGCTCCTATCCGTCCGG<br>CAACATCGCTTTGCCAGTG	95°C - 10 min;<br>40 cycles:<br>95°C – 30 s;<br>57°C – 1 min;<br>72°C – 30 s*<br>Melting	155 bp	Fraser, et al., 2017
	CAGTGGGACGACCACGAGGT	95°C - 10 min;		

PhoD	GAGGCCGATCGGCATGTCG	40 cycles:<br>95°C – 30 s;<br>58°C – 1 min;<br>72°C – 30 s*<br>Melting	371 bp	Sakurai et al., 2008

*Fluorescence reading

Table S2. Soil fertility and texture of the experimental areas (Farm 1 and Farm 2) for evaluating the effect of using a biofertilizer based on phosphate-solubilizing rhizobacteria (PSR) (*Bacillus velezensis* and *Lysinibacillus fusiformis*) on the promotion of soybean plant growth. Samples collected within (R) and between the planting rows (BR).

Area	Treatment	Clay	pH	Macronutrients					Micronutrients					H+Al	CEC	BS
				P	K	Ca	Mg	S	Bo	Cu	Fe	Mn	Zn			
		%	-	mg dm^{-3}		cmol$_c$.dm^{-3}		mg dm^{-3}						cmol dm^{-3}		%
Farm 1	With PSR (BR)	32.00	5.47	40.60	140.33	4.70	1.82	6.83	0.38	0.97	21.20	16.80	3.23	3.33	10.20	67.17
	Conventional (BR)	32.83	5.73	37.47	119.00	5.00	1.88	5.50	0.38	1.07	20.60	19.50	3.37	2.90	10.10	71.40
	With PSR (R)	34.50	5.80	42.10	133.33	4.87	1.86	9.66	0.48	0.93	20.60	17.80	3.40	3.30	10.40	68.33
	Conventional (R)	35.33	6.00	40.90	108.33	5.41	2.14	6.80	0.44	1.10	20.70	20.10	3.97	2.87	10.70	73.17
Farm 2	With PSR (BR)	40.33	4.87	44.57	102.33	4.48	1.05	13.60	0.39	1.00	29.90	20.00	4.73	5.10	10.90	53.30
	Conventional (BR)	43.67	5.00	55.67	107.00	5.59	1.36	25.43	0.46	1.03	28.70	22.40	6.60	4.70	11.90	60.40
	With PSR (R)	41.17	4.87	50.43	86.333	4.54	1.15	11.37	0.41	1.03	30.20	20.80	4.93	5.20	11.10	52.20
	Conventional (R)	34.50	4.93	38.83	114.00	4.42	1.04	8.87	0.38	0.97	24.8	15.90	5.73	4.93	10.70	53.87

CEC = cation exchange capacity; BS = Base saturation.
